# Tardive dyskinesia in Asia— current clinical practice and the role of neurologists in the care pathway

**DOI:** 10.3389/fneur.2024.1356761

**Published:** 2024-02-14

**Authors:** Roongroj Bhidayasiri, Onanong Phokaewvarangkul, Hui-Fang Shang, Thien Thien Lim, Jin Whan Cho, Pramod Kumar Pal, Hirohisa Watanabe

**Affiliations:** ^1^Chulalongkorn Centre of Excellence for Parkinson's Disease and Related Disorders, Department of Medicine, Faculty of Medicine, Chulalongkorn University and King Chulalongkorn Memorial Hospital, Thai Red Cross Society, Bangkok, Thailand; ^2^The Academy of Science, The Royal Society of Thailand, Bangkok, Thailand; ^3^Department of Neurology, West China Hospital of Sichuan University, Chengdu, Sichuan, China; ^4^Neurology Unit, Island Hospital, Georgetown, Penang, Malaysia; ^5^Department of Neurology, Samsung Medical Center, Sungkyunkwan University School of Medicine, Seoul, Republic of Korea; ^6^Department of Neurology, National Institute of Mental Health and Neurosciences, Bengaluru, India; ^7^Department of Neurology, Fujita Health University, Toyoake, Aichi, Japan

**Keywords:** tardive dyskinesia, Asia, treatment pathways, antipsychotic drugs, role of neurologists

## Abstract

Tardive dyskinesia (TD) is a movement disorder that can arise as a side effect of treatment with dopamine receptor-blocking agents (DRBAs), including antipsychotic drugs (APDs) used to manage psychotic illnesses. Second-generation APDs (SGAs) are often preferred to first-generation drugs due to their lower propensity to cause TD, however many SGAs-treated patients still develop the condition. Although TD is a global health concern, evidence regarding the occurrence of TD and how it is managed in Asian countries is currently limited. This article reports the results of a systematic review of the published literature on TD focusing on its prevalence, types of patients, knowledge of the condition, causative factors, and usual treatment pathways in clinical practice in Asian countries. Epidemiological data suggest that the prevalence of TD is increasing globally due to an overall rise in APD use, contributing factors being polypharmacy with multiple APDs, the use of higher than necessary doses, and off-label use for non-psychotic indications. Although exact prevalence figures for TD in Asian countries are difficult to define, there is a similar pattern of rising APD use which will result in increasing numbers of TD patients in this region. These issues need to be addressed and strategies developed to minimize TD risk and manage this disabling condition which impacts patients' quality of life and daily functioning. To date, both research into TD has been predominantly psychiatry focused and the perspectives from neurologists regarding the clinical management of this challenging condition are scarce. However, neurologists have an essential role in managing the movement disorders manifestations that characterize TD. Optimum management of TD, therefore, should ideally involve collaboration between psychiatrists and neurologists in joint care pathways, wherever practical. Collaborative pathways are proposed in this article, and the challenges that will need to be addressed in Asian countries to improve the care of people with TD are highlighted, with a focus on the neurologist's viewpoint and the implications for the management of TD globally.

## 1 Introduction

Tardive dyskinesia (TD) is one type of tardive syndrome, a group of iatrogenic movement disorders that have diverse clinical manifestations (1–3). The Diagnostic and Statistical Manual of Mental Disorders, Fifth Edition, Text Revision (DSM-V-TR) provides a standard definition of TD that is widely accepted by neurologists and psychiatrists and which specifies involuntary athetoid or choreiform movements lasting at least a few weeks developing in association with the use of a neuroleptic medication (dopamine receptor-blocking agent, DRBA) for a least a few months, and persisting beyond 4–8 weeks (4). In the DSM-IV-TR, TD refers to involuntary choreiform, athetoid, or rhythmic movements (lasting at least a few weeks) of the tongue, jaw, or extremities developing in association with the use of neuroleptic medication for at least a few months (may be for a shorter period of time in elderly persons) (5). All criteria require chronic DRBA use as an essential criterion for diagnosis. Dyskinesia that persists beyond 4–8 weeks is considered to be TD. TD is known to occur sometime after exposure to DRBA, “tardive” meaning “delay” and reflecting the timing of symptom onset. The DRBAs that give rise to TD include antipsychotic drugs (APDs) that are commonly prescribed for the treatment of psychiatric conditions, such as schizophrenia, bipolar disorder, and depression (6). Aside from APD, other types of DRBA have also been reported to be associated, including antiemetics, such as metoclopramide and promethazine, that are used for the treatment of gastrointestinal disorders (7). Alternative diagnostic specifications for TD have also been published, including the Schooler-Kane criteria, which requires exposure to APD, similar to DSM-V-TR, the absence of other causes, and a positive score on the Abnormal Involuntary Movements Scale (AIMS) (8). A 2 for one item and 1 in each of the other 6 body areas for a score of 8 is not TD, whereas two items each with a score of 2 but 0 elsewhere for a score of 4, would be positive for TD.

Although these standard definitions specify exposure to DRBAs, in particular APDs, as a fundamental diagnostic criterion for TD, other non-DRBA medications have been reported, albeit more rarely, to be associated with TD-like syndromes, including antidepressants, calcium channel blockers, anticholinergics, antiepileptics, and some anti-parkinsonian medications (7, 9–13). Many drugs used in psychiatric illnesses are known to have multiple pharmacological actions and it is possible that other therapeutic drug classes may have mild, or even unknown, DRBA, properties.

The uncontrollable, abnormal and repetitive hyperkinetic movements that characterize TD frequently occur in combination, and are usually irreversible (14–16). For the person experiencing these symptoms, TD can be severely disabling and is known to have a substantial impact on quality of life and social functioning (17), so recognition and timely management is critical.

From a historical perspective, reports of extrapyramidal side effects following treatment with APDs began to emerge in the 1950's in the field of psychiatry, not long after the introduction of these compounds into clinical practice. In 1957 in Germany, Schoenecker was the first to describe involuntary bucco-oral movements that persisted after these neuroleptic medications were reduced or discontinued and in 1964 Faurbye and colleagues in Denmark proposed the term “tardive dyskinesia” to movements developing after long-term use, hence the term “tardive” for delayed onset (16, 18, 19). In parallel with this, in Asia Japanese psychiatrists in 1966 described how people with psychiatric disorders could exhibit involuntary movements of their lips, eyelids, and neck muscles, naming them “Mogue-Mogue” movements, and suggested a possible relationship between these manifestations and APD use, although they could not confirm this link (20, 21). Subsequently, during the 1970's and 1980's, further reports were published by research teams in other Asian countries and regions including India, China, Taiwan and Singapore, investigating the prevalence of TD in schizophrenia patients, and raising concerns about its link with APD use (20).

APDs are generally categorized into two groups: the older “typical” or “first-generation” APDs (FGA) that were developed in the 1950's and the newer “atypical” or “second-generation” APDs (SGA) became available in the 1980's and are thought to have a lower propensity to cause TD when compared with FGA, due to their lower binding affinity for dopamine D2 receptors (2). Unsurprisingly, therefore, an analysis of global trends in APD use from 2004 to 2020 revealed that there was an increase over this time in the use of new SGAs rather than older FGAs, the most commonly prescribed agents being quetiapine, olanzapine, aripiprazole, and risperidone (22). The analysis also noted a relatively higher use of FGAs in lower-income countries. While the advent of SGAs has somewhat improved the risk of developing TD, the hope that they might mitigate the development of TD completely has not been realized (23). This was demonstrated in a meta-analysis published in 2017 of studies that reported figures for TD prevalence and APD. It found that while 30% of patients who were treated with FGAs subsequently developed at least mild TD, 20% of those treated with SGAs (but had received an FGA in the past) still went on to develop the condition (24).Notably, Among FGA-naive patients TD prevalence was lower at 7.2%. This problem is now compounded by the rising levels of use of APDs overall, which puts more patients at risk of developing the disorder.

Another critical point to consider in any discussion of TD is that management of this condition crosses two disciplines, since it is a neurological disorder arising as a side effect of treatment of a psychiatric condition. It would be expected, therefore, that optimum management of this disorder should ideally involve collaboration between psychiatrists and neurologists along the care pathway so that between them they can determine the right balance for each patient that can effectively manage the movement problems associated with TD while also ensuring that the underlying psychiatric condition is well-controlled and as far as possible does not deteriorate. This may not be practical or achievable in all care provision settings, but is something that should be aspired to. It is notable that if we look at trends in TD research, they are predominantly psychiatry focused. An analysis of publications over a time span of 54 years indicates that historically, most publications are from the areas of psychiatry and pharmacology, with less from the field of neurology and very few specifically relating to movement disorders, even though this is a core characteristic of this syndrome (25).

It is apparent from the global epidemiological data that TD is becoming a considerable public health concern. We can infer that this pattern of increasing TD prevalence due to rising worldwide APD use are likely to be reflected at regional and national levels too, however data to confirm this may be conflicting due to a range of different factors, including methodological issues and confirmation of definitive diagnosis, or may not be available at all.

Findings of the Research on Asian Psychotropic Prescription (REAP) study can provide some valuable insights relating to TD is Asia. REAP is a collaborative research initiative established in 2001 between Asian psychiatrists and pharmacologists to evaluate prescription patterns for APDs among Asian countries, how they differ across the region, and what factors drive these variations. Although their objective was not to investigate TD, their international surveys of patients with schizophrenia, depression and other disorders reveal important information about patterns of APD drug use that can help inform assessment of the occurrence of TD in the Asian region. In 2016, REAP undertook a cross-sectional survey across 15 Asian countries and territories collecting socio-demographic and clinical data on APD prescription patterns in adult schizophrenia patients using standardized procedures (26). The results confirmed that the trend of increasing APD use seen globally was also happening in Asia, although there was considerable variation between countries. Overall, there was a notable rise in the use of SGAs with 31.3 and 80.8% of the survey cohort receiving FGAs and SGAs, respectively. Outside of this information about prescription trends, however, robust and direct evidence regarding TD prevalence, causative agents, and management strategies in the region is currently very limited.

Our objective was to better understand the current situation regarding TD in Asia, specifically from a neurologist's perspective. To achieve this, we undertook a systematic review of the published literature on TD focusing on its prevalence, causative agents and risk factors, potential protective factors, knowledge of the condition, and usual treatment pathways in clinical practice in Asian countries.

## 2 Literature review methodology

A PubMed literature search was undertaken in April 2023 for published evidence regarding TD in Asian countries. The search was undertaken using PRISMA principles for systematic reviews (27) and used the search terms ((Asia) OR (Asian)) AND (((Tardive) OR (Tardive dyskinesia)) OR (Tardive syndrome)). For full details of the systematic review process see Supplementary material.

The search explored the following domains relating to TD: prevalence, clinical presentation, known and potential causative agents and risk factors, strategies to minimize development of TD, treatment options, and overall awareness of the condition, with the objective of understanding any unique characteristics, differences, or similarities between TD and its management in Asia compared with the situation in other world regions.

**Figure 1:**
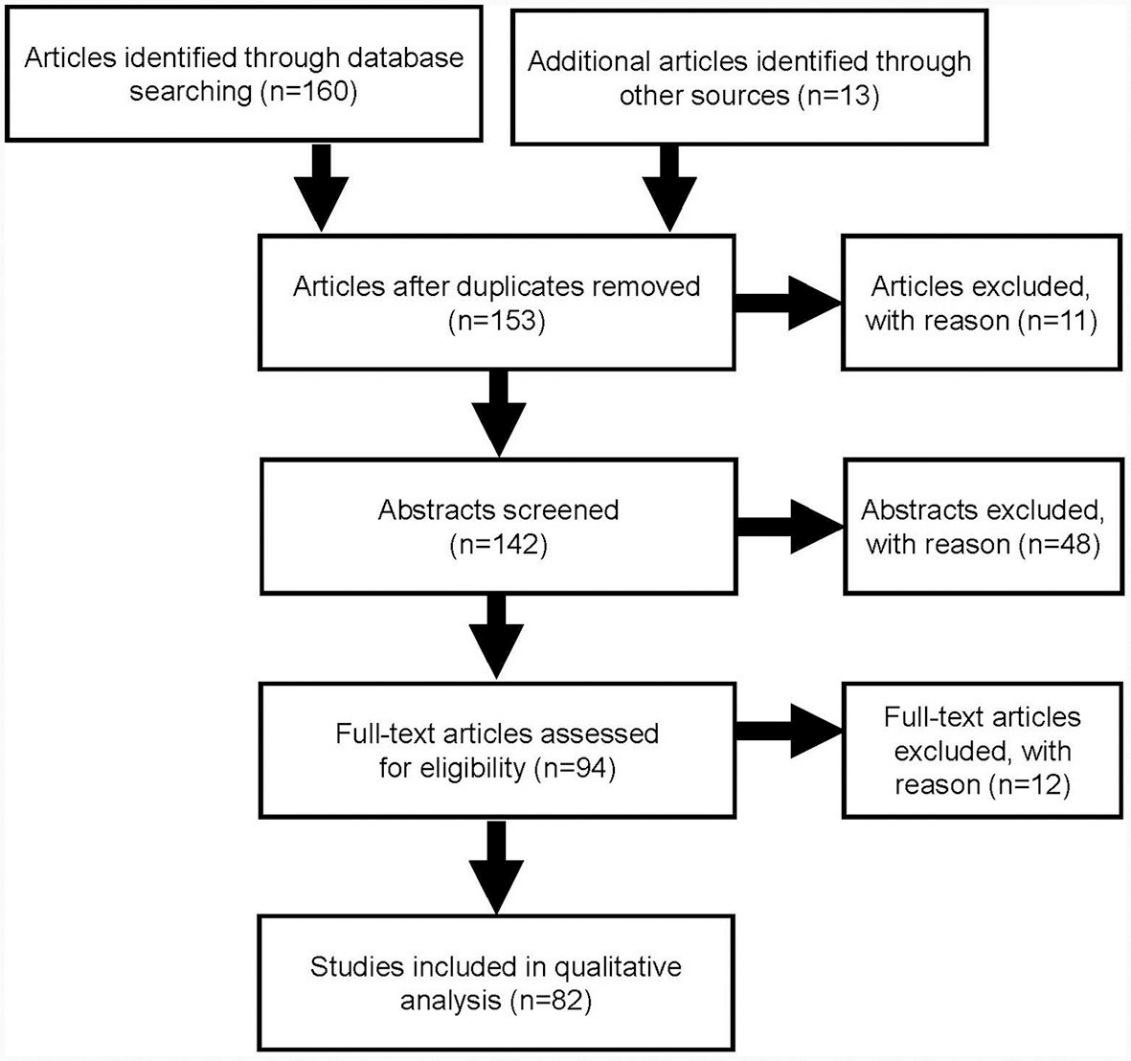
PRISMA literature search flow diagram.

The search identified 160 articles through database searching and a further 13 from other sources. After duplicates were removed and the articles screened, a total of 82 publications were included in this quantitative analysis (Figure 1).

## 3 Literature search results

Of the 82 studies identified for analysis, the majority were cross-sectional (42.7%), case-control (20.8%) or cohort (17.1%) studies (Table 1). Only two randomized, controlled trials were identified. Investigation of the medical disciplines of the teams who undertook the research in these studies revealed that the vast majority (74.4%) were undertaken by psychiatrists only with 9.7% being undertaken by a combination of psychiatrists and neurologists (Table 2). Only two studies (2.5%) were exclusively conducted by neurologists, highlighting the current limited engagement between the two disciplines and the current lack of involvement of neurologists in TD research and treatment.

**Table 1:** Breakdown of study types identified in the literature review (*n* = 82).

**Study design**	**Numbers of studies (%)**
Cross-sectional	35 (42.7%)
Case-control	17 (20.8%)
Cohort	14 (17.1%)
Meta-analysis	5 (6.1%)
Descriptive	3 (3.7%)
Retrospective	2 (2.4%)
Randomized, controlled trial	2 (2.4%)
Case series/report	2 (2.4%)
Other	2 (2.4%)
**All studies**	**82 (100%)**

**Table 2:** Breakdown of the different disciplines that undertook the research identified in the literature review (*n* = 82).

**Research conducted by (discipline)**	**Numbers of studies (%)**
Psychiatrist	61 (74.4%)
Psychiatrist and neurologist	8 (9.7%)
Pharmacologist	4 (4.9 %)
Medical geneticist	3 (3.7%)
Neurologist	2 (2.5%)
Psychiatrist and speech therapist	1 (1.2%)
Psychiatrist and orthopedic surgeon	1 (1.2%)
Endocrinologist	1 (1.2%)
Epidemiologist	1 (1.2%)
**All studies**	**82 (100%)**

The results of our literature search are discussed below comparing what has been found in Asian countries regarding TD and its management with global data and viewpoints.

## 4 Prevalence of TD

TD is known to have a prolonged clinical course and has an increased prevalence with longer exposure to DRBAs, therefore exact figures can be difficult to establish when comparing different reports. Results of a meta-analysis of 41 studies of subjects taking APDs suggest a global mean TD prevalence of 25.3% but this differs significantly amongst the major geographical regions, with figures of 22.3% reported for Europe, 31.3% for the USA, and 31.8% in other regions, such as Australia, Africa and the Middle East (24). The overall prevalence of TD in Asia has been estimated to be 17.3%, so lower than that found in the USA and in other Caucasian populations, suggesting that there may be ethnic and genetic factors underlying the risk of developing TD. However, while early review articles suggested that the prevalence of TD may be lower in Asia than in Western countries, more recent data have reported contradictory findings, so precise figures remain unclear (28).

When evaluating these TD prevalence figures, the possible confounding effects of availability of drugs and prescribing practices need to be considered. For example, a recent study at a single center in the UK found ethnic disparities in APD prescribing among a cohort of patients of mixed ethnicities, with black patients being less likely to be prescribed SGAs (29). Similarly, a systematic review of international studies found that Black and Hispanic schizophrenia patients in the UK and the USA are significantly less likely to receive clozapine than White/Caucasian patients (30). Ethnic differences have also been observed in the way some APD are metabolized, which will have an impact on selection of an appropriate drug dose (31, 32).

Of the 82 publications identified in our literature review, 32 studies reported figures for TD prevalence in their cohorts, although the majority were not specifically epidemiological studies (Table 3) (24, 33–39, 41–46, 48–62, 64, 65, 67). All but two studies were undertaken in schizophrenia patients. Of the remaining two, one reported data for patients taking antidepressants (59) and the other for patients with bipolar disorder (63). Most studies originated from either China or Japan. Studies covered the period from 1982 to 2022 and varied in design, setting, patient population, and in the assessment tools used to confirm a diagnosis of TD. The prevalence figures vary widely with low figures (0.57%) reported in a cross-sectional study of APD-treated schizophrenic patients at a single hospital in India with much higher figures of 44.5% reported in a study of male schizophrenic inpatients in China on long-term clozapine treatment (61). One study of 647 psychiatric inpatients in Japan found that 67% could be considered as having mild TD symptoms. A prospective study, also from Japan, of patients in 11 psychiatric centers also reported a much lower prevalence of TD (7.6%) than found in US and European populations (38). These wide-ranging prevalence figures highlight a limitation of this analysis in that many studies do not provide a medication history, which could bias the prevalence figures. The prevalence of TD in clozapine-treated patients reported in the study from China is high and inconsistent with previous studies. A contributor to this could be that clozapine is often used as an alternative therapy in treatment-resistant TD previously managed with other APDs that resulted in their TD, but which may only have emerged after switching to clozapine.

**Table 3:** Studies identified in the literature review that reported TD prevalence figures.

**Year**	**References**	**Country/region**	**Study details and patient population**	**TD prevalence**
1982	Doongaji et al. (33)	India	1,801 inpatients treated with APDs at a single psychiatric hospital.TD evaluated using AIMS.	Overall: 9.6% (173/1,801); men: 5% (108/1,141); women: 9.8% (65/660).
1986	Guy et al. (34)	International /Japan	International study of schizophrenia patients treated with APDs including one center in Japan (number of Japanese patients not specified). TD evaluated using AIMS.	13.6% of the total cohort (101/739). Specific data for Japanese patients not reported.
1987	Binder et al. (35)	Japan	126 patients treated with APDs, primarily for schizophrenia (94%), at 6 psychiatric hospitals. TD evaluated using AIMS.	Moderate or severe TD: 14.2% (18/126); mild TD: 20.6% (26/126).
1989	Ko et al. (36)	China	866 chronic schizophrenia patients (80.8% male, 19.2 % female) treated with APDs at a single psychiatric hospital. TD evaluated using AIMS.	8.4% (73/866).
1991a	Inada et al. (37)	Japan	716 patients treated with APDs at a single psychiatric hospital. TD evaluated using AIMS.	9.9% (71/716).
1991b	Inada et al. (38)	Japan	Prospective study of 1,595 patients treated with APDs at 11 psychiatric facilities. TD evaluated using AIMS.	7.6% at study entry; annual incidence rate: 3.7%; annual remission rate: 28.7%.
1991	Tan and Tay (39)	Singapore	514 patients aged ≥60 years of age treated with APDs, primarily for schizophrenia (74.7%), at a single psychiatric hospital. Cohort included Chinese, Indian and Malaysian patients.TD evaluated using the rating scale developed by Simpson et al. (40).	Inpatients: 31.5%; outpatients: 10.5%.
1992	Chiu et al. (41)	China	917 patients (503 male, 414 female) treated at a single psychiatric hospital; 94% had a history of APD use. TD evaluated using AIMS.	Overall: 9.3%; schizophrenia patients: 8.5%.
1992	Koshino et al. (42)	Japan	647 patients (361 male, 286 female) patients treated with APDs at a four psychiatric hospitals. TD evaluated using AIMS and Schooler and Kane criteria (8).	Overall: 22.3%; mild TD: 67.4%; moderate TD: 29.2%; severe TD: 3.5%.
1993	Chiu et al. (43)	China	Elderly patients treated at a psychogeriatric clinic (*n* = 160; 72 treated with APDs), a psychiatric hospital (*n* = 231; 202 treated with APDs), a geriatric day hospital (*n* = 68) and a senior citizen center (*n* = 113). TD evaluated using AIMS.	APD-treated patients: 25.9%.
1998	Ohmori et al. (44)	Japan	100 patients with schizophrenia treated with APDs. TD evaluated using AIMS.	24.2% (24/99).
2000	Kimura et al. (45)	Japan	333 inpatients with schizophrenia treated with APDs at three hospitals. TD evaluated using AIMS and Schooler and Kane criteria (8).	Overall: 18.6%; men 19.0%; women 18.2%.
2001	Chong et al. (46)	Singapore	607 patients with schizophrenia treated with APDs at a single psychiatric hospital. Cohort included Chinese, Indian and Malaysian patients.TD evaluated using AIMS and the Simpson-Angus Rating Scale (47).	39.9% (242/607).
2002	Chong et al. (48)	Singapore	333 inpatients with schizophrenia treated with APDs at three hospitals. TD evaluated using AIMS.	Overall: 39.3%; Chinese: 40.6%, Malaysian: 29.0%.
2003	Leung et al. (49)	China	225 inpatients with chronic schizophrenia treated with APDs at a single psychiatric hospital. TD evaluated using AIMS and the Simpson-Angus Rating Scale (47).	6.7% (15/225).
2004	Bhatia et al. (50)	India	334 outpatients with schizophrenia. TD evaluated using AIMS and the Simpson-Angus Rating Scale (47)	28.7% (96/334); of these, 39.6% had received only FGAs, 47.9% had received only SGAs and 12.5% had received both.
2005	Tiwari et al. (51)	India	Retrospective, cross-sectional study of 335 patients with chronic schizophrenia at a single hospital. TD evaluated using AIMS.	28.7% (96/335); of these, 29.2% (28/96) had received only FGAs, 24.0% (23/96) had received only SGAs and 46.9% (45/96) had received both.
2006	Kasper et al. (52)	East Asia	Study population was part of the Intercontinental Schizophrenia Outpatients Health Outcomes study (IC-SOHO) and included 1,167 patients from East Asia. TD symptomatology ranked by the treating physician using a 4-point scale.	Overall: 8.9%; East Asian countries: 8.5%.
2006	Hori et al. (53)	Japan	222 inpatients with chronic schizophrenia treated with APDs. TD evaluated using AIMS.	20.7% (46/222).
2009	Chong et al. (54)	Singapore	608 patients with schizophrenia (76.7% male) treated with APDs at a single psychiatric hospital. Cohort included Chinese, Indian and Malaysian patients.	Mild TD: 7.7%; definite TD: 39.6%.
2009	Go et al. (55)	Philippines	227 patients with schizophrenia treated with APDs at a single mental health center. TD evaluated using AIMS and the Simpson-Angus Rating Scale (47)	20.3% (46/227).
2009	Zhang et al. (56)	China	Cross-sectional naturalistic study of 522 inpatients with schizophrenia treated with APDs at a single psychiatric hospital. TD evaluated using AIMS.	Overall: 33.7%; men: 39.2%, women: 22.4%.
2009	Li et al. (57)	China	101 patients with schizophrenia treated with clozapine as a primary APD at two psychiatric hospitals. A diagnosis of TD was a score of ≥3 on at least one item or a score of 2 on two or more items of the Extrapyramidal Symptom Rating Scale (ESRS).	3.96% (4/101).
2011	Zhang et al. (58)	China	764 male patients with chronic schizophrenia treated with APDs at a single mental health center. TD evaluated using AIMS.	Overall: 40.1%; smokers: 41% (237/578); non-smokers: 37% (69/186).
2013	Lee et al. (59)	Taiwan	Retrospective chart review of 158 patients who had received antidepressants at a single hospital. The presence of tardive syndromes (TS) was assessed by a research psychiatrist using the Extrapyramidal Symptom Rating Scale (ESRS).	Overall: 14% (22/158) had some form of TS: TD prevalence: 3.2 %.
2014	Achalia et al. (60)	India	Cross-sectional study of 160 patients with schizophrenia treated with APDs at a single tertiary care center. TD evaluated using AIMS and Schooler and Kane criteria (8).	Probable TD: 26.4% (42/160).
2014	Ye et al. (61)	China	Cross-sectional naturalistic study of 584 male schizophrenia patients treated with APDS at 2 hospitals. TD evaluated using AIMS and Schooler and Kane criteria (8).	Overall: 44.5%; FGA-treated patients: 38.7% (94/243); clozapine-treated patients: 48.7% (166/341).
2017	Carbon et al. (24)	Multiple Asian countries	A meta-analysis of published data from 2000 on TD prevalence in psychiatric patients treated with FGAs or SGAs. Inclusion criteria specified that TD was assessed using a standardized rating scale.	Global: 25.3%; Asian countries: 17.3%.
2018	Desai et al. (62)	India	Cross-sectional study of 584 patients taking APDs at a single hospital. TD evaluated using AIMS and Schooler and Kane criteria (8)	Overall prevalence of movement disorders: 5.67%; TD: 0.57%.
2018	Rajan et al. (63)	India	Cross-sectional study of 170 patients with bipolar disorder treated with APDs at a single tertiary care center. TD evaluated using AIMS and Schooler and Kane criteria (8).	Overall: 10.6% (18/170); men: 14.7%, women: 4.4%.
2021	Uludag et al. (64)	China	901 inpatients with chronic schizophrenia treated with APDs at two hospitals between 2008 and 2011. TD evaluated using AIMS.	Overall: 36%; men: 41.4%, women: 12.1%.
2022	Liang et al. (65)	China	655 patients with chronic schizophrenia treated with APDs at two hospitals. TD evaluated using AIMS.	Overall: 41.1%; men: 42.9%, women: 28.4%.

AIMS, Abnormal Involuntary Movement Scale (66); APDs, antipsychotic drugs; FGAs, first-generation antipsychotic drugs; REAP, Research on Asian Psychotropic Prescription Patterns; SGAs, second-generation antipsychotic drugs.

Based on the wide-ranging figures reported, it is not possible to confirm definitively that TD prevalence is lower in Asia than in some other world regions (22–32%), in fact some studies reported a higher prevalence. However, it is important to note that the variability these in TD prevalence figures may be due in part to differences in study design, data collection and TD assessment methods and heterogeneity of patient populations across regions and countries, coupled with the considerable challenges of undertaking robust epidemiological studies. Variations in prescribing patterns in these regions, types of APD used and dosing schedules also need to be considered.

## 5 Causative agents and risk factors for the development of TD

Various risk factors for developing TD have now been identified (68–70), and for the purposes of their management, are grouped into two broad categories: modifiable and non-modifiable. While DRBAs, including APDs, are known to be causative agents in the development of TD and are an inherent part of its definition, they can also be considered as risk factors depending on how they are used (e.g., dose, frequency, and duration). Modifiable risk factors include the use of APDs and some other therapeutic drugs, and the presence of certain comorbid conditions, such as diabetes mellitus, that can be controlled with suitable therapeutic management (68, 71). Similarly, smoking as well as alcohol and substance abuse, are also known to increase TD risk, but have the potential to be modified with lifestyle changes. Non-modifiable risk factors include those inherent factors such as sex, age, ethnicity and genetic make-up that cannot be changed. However, it is important to note modifiable and non-modifiable risk factors are not mutually exclusive—some non-modifiable factors can influence modifiable factors, for example TD patients of Asian heritage (a non-modifiable factor) are more likely to be affected by diabetes mellitus (a modifiable factor).

### 5.1 Modifiable risk factors

#### 5.1.1 APD use

As previously discussed, the association between APD use and the development of TD is well-known in the global literature and is an example of a potentially modifiable risk factor. Such modifications often include the use of SGAs in preference to FGAs, and reducing the dose and duration of exposure to APDs wherever possible (68, 71). However, it should be noted that data supporting the suggestion that SGA are less likely to cause TD than FGA are subject to debate, and while the general consensus from experts is that SGA are safer, not all data support this contention. In fact, analysis of data from the Clinical Antipsychotic Trials in Intervention Effectiveness (CATIE) study of patients with schizophrenia found no significant differences in the incidence of TD among patients in receiving the FGA perphenazine or four different SGAs (72).

In Asia, to the best of our knowledge, the first description of a possible link between APD use and TD was reported in Japan in 1966 (21). Since that time, and in common with trends in other world regions, Asian countries have seen a rise in the use of APDs (73). The 2016 REAP study reported that the most commonly-prescribed antipsychotics for schizophrenia patients in Asia were haloperidol and risperidone (26). Overall, nearly one-third (31.3%) received FGAs while 80.8% received the newer SGAs.

However, a further analysis of data from REAP looking at 15 Asian countries found a high prevalence of APD polypharmacy (simultaneous prescribing of more than one APD) with subjects often treated with higher than recommended doses (74). Similarly, a study of older Asian patients with schizophrenia in six Asian countries and regions (China, Hong Kong, Japan, Korea, Singapore and Taiwan) from 2001 to 2009 found that high doses of APDs were used in more than one-third of patients (75). Added to this, a “real-world” survey from China found that off-label use of APD is common among psychiatric inpatients (76). APDs were prescribed for a wide spectrum of non-psychotic psychiatric disorders with the rate being the highest among patients with dissociative disorders, organic mental disorders, dementia, obsessive-compulsive disorder, depression, anxiety, and insomnia. The most commonly used antipsychotics used off-label were the SGAs olanzapine, quetiapine and risperidone. Similar results have been reported in a systematic review of the global literature regarding off-label APD use in adults, children and the elderly (77). Across 77 studies, off-label prescribing constituted 40–75% of APD prescriptions for adults, the main indications being mood disorders, anxiety disorders, insomnia and agitation. In common with the data from China, quetiapine was the most frequently prescribed off -label APD. Overall, these observations of higher than necessary exposure to APDs and considerable off-label use are likely to put more patients at unnecessary risk of developing TD so need to be monitored and managed.

The studies identified in our literature review confirmed that longer duration of APD exposure is a risk factor for TD development (55, 56, 60, 63). Higher cumulative APD dose (55, 60), the use of typical, FGAs (24, 56, 63, 78) and intermittent treatment with APDs (60) were also identified as risk factors. In contrast with these reports and the global published literature, a few studies have suggested a link between TD development and lower current dosage of APD (41–43, 48, 79). However, drug formulation also seems to have an influence. One study from Japan that included 8,425 patients found less reporting of TD when they were treated with long-acting injectable APDs, particularly long-acting SGAs, vs. the equivalent oral APDs (80). These findings are in line with those reported in a US study of chronic treatment with long-acting, injectable risperidone which found a low 1-year rate of treatment-emergent TD (1.19%) among 530 schizophrenia patients (81).

In terms of the specific drugs that have been linked to the development of TD, a wide range of APDs have been identified in studies from Asia (Table 4). Those most commonly noted in the studies identified in the literature search as being associated with TD development were the FGAs chlorpromazine and haloperidol (possibly reflecting their established use, wider availability and lower cost), closely followed by the SGAs clozapine and risperidone.

**Table 4:** Antipsychotics and other drugs associated with the development of TD reported in publications identified in the literature review.

**First-generation (typical) APDs**	**Second-generation (atypical) APDs**	**Other drug classes**
Haloperidol, oral Chlorpromazine Sulpiride Perphenazine Trifluoperazine Levomepromazine Fluphenazine, oral Thioridazine Bromperidol Loxapine Pipotiazine palmitate injection Flupenthixol decanoate, depot Fluphenazine decanoate, depot Zuclopenthixol decanoate, depot Chlorprothixene Fluphenazine, injection Propericiazine Promazine Pipamperone Haloperidol, injection	Clozapine Risperidone Olanzapine Quetiapine Aripiprazole Mosapramine Zotepine Clocapramine Sertindole Sultopride Clotiapine Ziprasidone Amisulpride	Promethazine, an antihistamine

Drugs are listed in order of the frequency of reports among the studies identified in the literature search.

#### 5.1.2 Antidepressants

Antidepressant-induced movement disorders have been described in the literature, although they are a relatively rare occurrence and, unlike many cases of TD, are usually reversible (10, 11).

Currently, the most commonly prescribed antidepressants are selective serotonin reuptake inhibitors (SSRIs) and agents that inhibit the reuptake of norepinephrine, or both serotonin and norepinephrine (SNRIs) (82). More recently, to help manage residual depressive symptoms that can occur despite treatment with SSRIs and SNRIs and that may be related to dopaminergic pathways, dual reuptake inhibitors of norepinephrine and dopamine (NDRIs), such as bupropion, have been developed (83). Triple reuptake inhibitors (TRIs) that act on serotonin, norepinephrine and dopamine transporters have also been investigated for their therapeutic potential in depression (84).

Modulation of monoaminergic neurotransmission in the brain's frontal cortex and in other corticolimbic structures is thought to play an important role in the actions of both APDs and antidepressants, but studies have revealed that there is a complex interplay of reciprocal interactions between these different neurotransmitter pathways (85). In addition, while different antidepressants vary in their potency for inhibition of the reuptake of monoamine neurotransmitters to exert their clinical effect, they also exhibit a range of affinities for blocking various neurotransmitter receptors, including dopamine D_2_ receptors (12).

Since they are not related to DRBA exposure, the movement disorders induced by antidepressants are not the same as TD, but can be classified as TD-like syndromes. An analysis of the World Health Organization's Pharmacovigilance database found that citalopram, paroxetine, duloxetine and mirtazapine were the antidepressants most frequently associated with movement disorders (86). The serotonin-norepinephrine reuptake inhibitor (SNRI), venlafaxine, was particularly associated with TD-like syndromes.

In Asia, a retrospective, epidemiological study from Taiwan also reported that several antidepressant medications may increase the risk of developing tardive syndromes (59). Agents include the selective serotonin reuptake inhibitors (SSRIs) fluoxetine, paroxetine, escitalopram, the SNRIs venlafaxine and duloxetine, the tricyclic antidepressants (TCAs) amitriptyline and trazodone, as well as mirtazapine and bupropion. The study included 158 subjects of which 22 (14.0%) were found to have at least one tardive syndrome. Tardive dystonia was the predominant form; however, a TD-like syndrome was the next most common occurring in 3.2% of these subjects. The authors confirmed that study subjects had been receiving antidepressants for over 6 months but no other agents that might have caused involuntary movements, and possible confounding medical conditions had been ruled out. In this particular study, the authors state that subjects did not have prior exposure to DRBAs. However, in cases where DRBA have been used prior to antidepressants, this may result in a “priming” effect. In addition, some antidepressants may have other effects, such as blocking the reuptake of dopamine or blocking dopamine receptors (12, 13).

#### 5.1.3 Other modifiable risk factors

Data from the global literature suggests that TD is more prevalent in those who have certain coexisting medical conditions, including diabetes, some neuropsychiatric disorders (schizophrenia, cognitive dysfunction), organic brain damage or alcohol dependence (68–70).

Studies from Asian countries reflect these findings, reporting an association between comorbid diabetes (87) or cognitive deficit (63) and TD. In the case of diabetes, it should be noted that it is also an adverse effect of sustained APD treatment, so can commonly occur as a co-morbid disorder. Diabetic gastroparesis is often treated with metoclopramide, a DRBA, which may contribute to this association. However, the association of TD with both diabetes and cognitive decline may be confounded by the fact that they are both associated with aging, as is TD (88).

Factors that are reported to occur more commonly in patients with TD in Asia include a long duration of psychiatric illness (24, 55, 56, 58, 60, 63–65, 87, 89) as well as baseline parkinsonism, a greater severity of extrapyramidal symptoms, or a diagnosis of schizoaffective disorder (24, 50, 90). In addition, some studies have reported fewer years of education in their TD patient cohorts compared to those without TD (64, 87).

### 5.2 Non-modifiable risk factors

Much of the interindividual variability in response to APDs and the profile of side effects experienced are thought to be due to genetic factors that impact the pharmacokinetics and pharmacodynamics of these drugs (91). Therefore, genetic differences between individuals and populations are important to consider (31). The concept of pharmacogenomics is now being investigated in relation to APD prescribing in order to inform the selection of the most suitable therapeutic agent based on the patient's genetic information (92) and the link between a range of different genetic factors and the risk of developing TD is the subject of considerable research (93). These include dopamine and enzymes that metabolize it (such as dopamine beta-hydroxylase), serotonin, and the cytochrome P450 (CYP) family of enzymes in the liver that metabolize APDs and also many antidepressant medications, in particular CYP1A2, CYP2D6, CYP3A4, with CYP2C19, each of which can exhibit various polymorphisms that result in different phenotypes

Potential genetic factors identified in our literature review that have been shown to be associated with TD in Asian studies, or where a lack an association has been observed, are summarized in Table 5. Currently the data are limited but include several studies on CYP enzyme and dopamine receptor gene polymorphisms, plus individual studies on a range of other genetic factors including enzymes and signaling factors. One study from China found a higher frequency of the haplotype T-4b-Glu, a genetic variant of the endothelial nitric oxide synthase (NOS3) gene, in non-TD than in TD subjects, suggesting it may have a potential protective effect against the development of TD. Further cross-cultural studies are needed to consolidate these findings and determine how this range of genetic factors might influence the efficacy and tolerability of APDs in populations with different ethnicities.

**Table 5:** Possible genetic risk factors for the development of TD reported in publications identified in the literature review.

**Year**	**References**	**Country/region**	**Study details and potential genetic risk factors**
Cytochrome P450 (CYP) enzyme polymorphisms			
1998	Ohmori et al. (44)	Japan	100 schizophrenia patients. TD evaluated using AIMS. CYP2D6^*^10 genotype showed a significant association with total AIMS scores and a modest association with TD occurrence.
2001	Lam et al. (94)	China	38 schizophrenia patients (22 men, 16 women). TD evaluated using AIMS. No significant association with CYP2D6^*^10 genotypes and TD found in men, but significant increase in the frequency of the CYP2D6^*^10 allele found in women.
2004	Liou et al. (95)	China	216 schizophrenia patients. TD evaluated using AIMS. CYP2D6^*^10 C188T polymorphism may be associated with susceptibility to TD induced by FGAs, especially in male patients, and it correlated with AIMS scores in TD patients.
2005	Tiwari et al. (51)	India	335 chronic schizophrenia patients. TD evaluated using AIMS. No association was found between CYP3A4^*^1B and CYP2D6^*^4 in susceptibility to TD. Trend toward increased severity of TD in patients heterozygous for the CYP2D6^*^4 variant.
Dopamine receptor gene polymorphisms			
2002	Woo et al. (96)	South Korea	54 schizophrenic patients without TD and 59 with TD. TD confirmed using AIMS. DRD3gly-gly polymorphism of the dopamine D3 receptor may be involved in the pathogenesis of TD.
2003	Chong et al. (97)	China	117 schizophrenic patients with TD compared with 200 patients without TD. TD confirmed using AIMS. No association found between polymorphism of the dopamine D2 receptor gene and TD but an increased risk of developing TD was observed in patients with the dopamine D3 receptor serine/serine genotype.
Other genetic factors			
2000	Kimura et al. (45)	Japan	333 schizophrenic patients; 62 with TD (31 men, 31 women). TD evaluated using Schooler and Kane criteria (8). No significant differences in apolipoprotein E (ApoE) 4 allelic frequency found between TD and non-TD patients. However, allele frequency was significantly lower in female TD patients than in male TD patients.
2004	Pae et al. (98)	South Korea	107 schizophrenia inpatients and 106 healthy controls. TD evaluated using AIMS and Schooler and Kane criteria (8). Quinone oxidoreductase gene (NQO1) polymorphism (609C/T) may confer susceptibility to the development of TD.
2006	Liou et al. (99)	China	282 schizophrenic patients treated with FGAs; 153 with TD, 129 without TD. TD confirmed using AIMS. Frequency of haplotype T-4b-Glu (a variant of the endothelial nitric oxide synthase [NOS3] gene) was significantly higher in non-TD than in TD group, suggesting a protective effect and the potential involvement of free radicals and oxidative stress in the pathogenesis of TD.
2008	Liou et al. (100)	China	Case-controlled study of 381 schizophrenic patients (228 with TD, 153 without TD). A positive association was found between the single nucleotide polymorphism rs1045280 on the ARRB2 (ß-arrestin 2) gene and TD in schizophrenic patients.
2010	Syu et al. (101)	Japan	86 schizophrenic patients with TD, 186 without TD). TD evaluated using Schooler and Kane criteria (8). Single nucleotide polymorphisms in the (heparan sulfate proteoglycan 2) (HSPG2) gene may be involved in APD-induced TD and higher expression of HSPG2, even after APD treatment, may be associated with TD susceptibility.
2013	Sun et al. (102)	China	412 schizophrenic patients without TD and 372 with TD. TD confirmed using AIMS. Allele and genotype frequencies of rs1800872 and rs72393728 (located on the promoter of interleukin-10 and dopamine beta-hydroxylase gene, respectively) did not significantly differ between patients with and without TD.
2013	Zhou et al. (103)	China	747 schizophrenic inpatients (619 men, 128 women). TD evaluated using AIMS. Dopamine beta-hydroxylase gene polymorphism 5′-Ins/Del is not associated with susceptibility to TD.
2014	Miura et al. (104)	Multiple Asian countries	Systematic review and a meta-analysis of the effects of brain-derived neurotrophic factor (BDNF) Val66Met polymorphism on APD-induced TD in Asian and Caucasian patients. No significant association found between BDNF Val66Met polymorphism and TD or AIMS scores across all patients, however, BDNF Val66Met polymorphism thought to affect severity and, possibly, TD development in Caucasians.
2021	Lim et al. (89)	East Asia	Genome-wide association study of TD using a meta-analysis of samples from schizophrenic patients of East-Asian, European, and African American ancestry. TD evaluated using AIMS. Single nucleotide polymorphisms in TNFRSF1B (a protein-coding gene that mediates antiapoptotic signaling) and CALCOCO1 (a protein-coding gene involved in the activation of transcriptional activities) were associated with a 3-fold increase in TD risk outside of other clinical risk factors.

AIMS, Abnormal Involuntary Movement Scale (66); APDs, antipsychotic drugs; FGAs, first-generation antipsychotic drugs.

Older age, female sex, the presence of coexisting medical conditions, such as diabetes and certain neurological disorders (schizophrenia, cognitive dysfunction, brain damage) are also factors known to be associated with an increased risk of developing TD (68–70). Some case reports have also suggested that patients with human immunodeficiency virus (HIV) infection or acquired immunodeficiency syndrome (AIDS) have a greater propensity to develop certain movement disorders, including TD, when taking neuroleptic medications (105–107).

Analysis of the literature for Asian countries found a large number of studies reporting that older age is a risk factor for the development of TD (24, 35, 38, 41, 43, 48–50, 53, 55, 56, 58, 60, 63–65, 87, 89, 108), in line with what has been seen in the global literature.

A predominance of TD in women was also found in two reports from Asia: a cross-sectional study of schizophrenic patients in the Philippines (55) and a retrospective study of elderly psychiatric patients at a single center in Singapore (39). In contrast, several studies found the opposite, reporting a higher prevalence of TD in male patients (50, 63, 64, 87, 89, 90, 108). In addition, sex differences in the clinical response to APDs and the side effects experienced have been reported in a study of schizophrenic patients in Pakistan (109). Data from the REAP study on APD prescribing patterns and side effects for 6,441 Asian patients with schizophrenia found that males used higher APD doses, fewer prescriptions for second-generation APDs and a higher degree of APD polypharmacy than females (110). It is possible that these sex disparities might reflect treatment inequalities in these countries, for example if women have less access to medical care and specialist assessments than men, this might put them at greater risk of TD. While possible treatment inequalities for TD are suggested by these findings, this issue has been less well-studied in TD than in other movement disorders and so results are currently equivocal.

## 6 Strategies for minimizing and managing TD

### 6.1 Minimizing the risk of developing TD—addressing prescribing practices

There are strategies available to minimize the risk of developing TD among people who are prescribed DRBAs. A critical factor in this is vigilance on the part of both the treating clinician and the patient for signs and symptoms of TD, as well as good communication to assess, report, and discuss these symptoms.

RE-KINECT was a prospective study that evaluated the presence and healthcare burden of TD in a “real-world” population of 739 APD-treated patients. The results confirmed that TD occurs frequently-−25% of the cohort had involuntary movements consistent with TD—and can significantly reduce patients' quality of life, physical wellness and social functioning (17, 111). Notably, however, the RE-KINECT results also showed that clinician-rated severity of TD did not always correlate with patient perceptions of the significance of TD (17). These findings reinforce the importance of clinicians routinely gathering information from patients about any symptoms, for example by using self-assessment screening tools, and asking about the impact these are having on their daily lives. However, data from a recent US survey of electronic medical records at a large community mental health treatment center suggest that routine screening for abnormal involuntary movements in APD-treated patients using validated scales, such as AIMS, can be low (112). Another US study found that implementing a 1-h training course for clinicians regarding AIMS screening for TD significantly improved the use of the tool and subsequent documentation in patients' records (113). Recognizing that the impact of TD may not always depend solely on the severity of TD symptoms, a new rating scale—Impact-TD—has been developed to assess the impact of the condition on patients' daily functioning which can be implemented easily in a clinical practice setting to provide further insights for the clinician (114). Although telehealth is something that is becoming increasingly used in many fields of medicine, a report from panel of experts comprising both neurologists and psychiatrists, conclude that while it was a useful adjunctive tool for the assessment of TD, it was not an adequate substitute for in-person assessment (115).

To reduce the risk of developing TD when prescribing APDs, ideally clinicians should employ the principle of “minimum dose utilization,” and select a suitable dose that achieves sufficient clinical benefit while at the same time minimizing adverse effects. However, this approach is often confounded by the fact that clinicians may prescribe multiple APDs at a time (polypharmacy) and use higher doses than necessary (116), a problem that has also been identified in Asian countries (74–76).

That said, it should be recognized that prescribing clinicians may be under certain pressures to maintain APD dosages unnecessarily high, including the desire to achieve predictable benefit quickly for their patients, or to contain disruptive behaviors and avoid psychiatric emergencies. Similarly, they are unlikely to want to “rock the boat” by changing the treatment regimen once their patient's condition has settled. To help clinicians navigate this complex situation, evidence-based treatment guidelines for TD have been published along with a practical treatment algorithm (117, 118). Downward titration to the minimum effective and maximally tolerated dose is an essential component of maintenance management, and failure to undertake this vital step may result in long-term exposure at high APD dosages.

It is worth noting that antipsychotic drugs are primarily indicated for the treatment of acute and maintenance psychotic disorders such as schizophrenia and bipolar disorder. They are not intended for use as hypnotics or anxiolytics. In the US, aripiprazole and brexpiprazole are both approved for treating “refractory depression” without psychotic features, and that olanzapine is approved to treat depression in bipolar patients, whether psychotic or not. Despite these specifications, as already described, a contributing factor to the rise in APD use seems to be their increased off-label use for non-psychotic indications, such as dementia, insomnia, obsessive–compulsive disorder, and generalized anxiety disorder (119). Survey data from England over the period from 2000 to 2014 found that while psychotic symptoms in the population had increased only slightly over this period, prescription of APDs had doubled (120).

In order to reduce the overall burden of TD, it is critical that prescribing patterns are addressed. However, they are not uniform across countries and world regions due to variations in availability, approved indications, access to these treatments and usual clinical practice. In terms of incidence and prevalence of APD use, a study examining trends in APD prescribing over the period from 2002 to 2014 in Taiwan, Hong Kong, Japan, and the USA found that haloperidol (an FGA) and quetiapine (an SGA) and were most common APDs prescribed in the USA and Hong Kong, and there was a trend for increased quetiapine use in these countries, as well as in Taiwan (73). In all countries the younger patients were more likely to be prescribed an SGA than older patients.

Authors of a recent systematic review of data relating to APD side effects suggest that a more evidence-based and personalized approach to APD prescribing is needed (121). Assessing and managing APD side effects, including TD, can be a complex and nuanced process in patients with ongoing psychiatric conditions, and although guidelines recommend discussing possible side effects with patients when prescribing and monitoring for them, there is little guidance available to navigate this process. They have developed a digital tool that supports navigation of a side effects database while incorporating user feedback to help facilitate clinical care.

### 6.2 Managing established TD—treatment pathways

#### 6.2.1 Establishing a differential diagnosis

Before a suitable treatment plan can be put in place, it is essential to establish an accurate differential diagnosis of TD (122). This can be challenging as the symptoms can overlap with those of other movement disorders, or phenomenologies arising from advancing age or mental illness (123). Added to this, there is considerable inter-patient variability in the duration of the delay between starting DRBAs and presenting with TD. Studies of Asian patients have reported the development of TD within 1 month to over 12 months (33, 36, 38, 79, 95, 96, 124), some between 5 and 10 years (125, 126), and others after more than 10 years of starting DRBAs (53, 125, 126).

As discussed earlier, globally the most commonly used diagnostic criteria for TD is the Diagnostic and Statistical Manual of Mental Disorders, Fifth Edition, Text Revision (DSM-V-TR) (4) and the Schooler-Kane criteria (8). A recent modified Delphi Consensus study of the screening, diagnosis and treatment of TD by a panel of experts comprising psychiatrists and neurologists agreed that the DSM-V-TR and Schooler-Kane criteria were generally appropriate for diagnosing TD, however there was a need for improved standardized criteria that could be applied more easily in routine clinical practice (127). In the case of the Schooler-Kane criteria, it has been proposed that specification of “a positive finding” on the AIMS requiring at least moderate in one item or mild in two items may be too high a threshold to diagnose TD, and that a lower threshold of at least one item rated mild on the AIMS may be sufficient to consider TD in clinical practice (127, 128).

AIMS is the most common rating scale used in TD and comprises 14 items: 1–4 assess orofacial movements; 5–7 deal with extremity and trunk movements; items 8–10 deal with global severity, patient's awareness and associated distress; items 11–14 are related to dental status and sleep (66). However, although AIMS is effective, valid and reliable, it has certain clinimetric limitations and does not capture different tardive phenotypes or sensory symptoms which are common in TD such as pain and discomfort (129–131). In addition, it does not evaluate symptom fluctuations. It has been recommended therefore that AIMS is revised to capture the broad range of TD syndromes and phenomenologies, while ensuring it retains its clinical utility as a practical tool for differential diagnosis (131).

Since AIMS does not adequately distinguish between the many different types of abnormal movements that patients can present with, this may partly explain why psychiatrists tend to group the various tardive variants into a single category. TD is often used as an umbrella term to refer to all abnormal movements, not just the predominantly oro-bucco-lingual dyskinesia of TD, whereas in fact the abnormal movements that characterize tardive dystonia and tardive akathisia, for example, present very differently to TD, affect different types of patients, and require different treatment approaches.

Another factor that may impede an accurate differential diagnosis of TD is that the terminology and classification of movement disorders has evolved in the field of neurology (132). However, the broad term “extrapyramidal symptoms” is still commonly used in psychiatry when describing the TD clinical picture whereas it actually has a very imprecise meaning. In neurology, it is no longer used in relation to movement disorders as it does not reflect clinical symptomatology, but is anatomically based.

#### 6.2.2 Treatment options

TD is associated with a substantial economic burden in terms of increased health care resource utilization, including more frequent inpatient hospital admissions (133), so timely and effective treatment are essential. In the Asian region, studies from China and Japan have also confirmed higher rates and duration of hospitalizations in TD patients compared to those without TD (35, 56, 58, 64, 65). Two studies from China report that the presence of TD is also associated with a higher degree of cognitive impairment which has implications for healthcare provision (65, 67). Importantly, mortality in Asian patients has been shown to increase with severity of TD: one study reported it to be 7.2% in those without TD rising to 10.6 and 18.3% in those with mild and definite TD, respectively (54). However, as discussed above for the association of TD with diabetes and cognitive impairment, mortality may be confounded by the increased age and declining health of people with TD rather than being an effect of TD itself.

Withdrawal of APD medication or switching from an FGA to an SGA have previously been suggested as approaches to reduce TD, but there is limited high-level evidence to support these strategies (117, 118). Globally, a range of different agents have been evaluated for the treatment of TD with varying degrees of success and often with equivocal results (134).

The recent US Food and Drug Administration approval of two novel vesicular monoamine transporter 2 (VMAT2) inhibitors, deutetrabenazine and valbenazine, for TD have changed the treatment landscape and offered new hope for the effective management of TD (135–138), however they are not yet available in all countries. An older VMAT2 inhibitor, tetrabenazine, is currently approved for the treatment of moderate to severe TD in some countries (138).

Two 12-week double-blind, randomized placebo-controlled trials (RCTs) of have been undertaken with deutetrabenazine, ARM-TD and AIM-TD, and four studies with valbenazine, KINECT 1–4 (KINECT 1– 3 are RCTs; KINECT 4 is an open-label study; KINECT 1 and 3 have open-label extension studies). A meta-analysis of data from these studies reported that both deutetrabenazine (24–48 mg per day) and valbenazine (40 or 80 mg per day) are effective in treating TD, both acutely and long-term, with significant improvements in AIMS scores from baseline vs. placebo (136). However, improvement in patient global impression of change (PGIC) and clinical global impression for deutetrabenazine and PGIC for valbenazine did not reach statistical significance. Further analysis of data for ARM-TD and AIM-TD found that for deutetrabenazine the minimal clinically important change in total motor AIMS score was a reduction of 2 (139).

As a result, the latest evidence-based guidance from a systematic review now includes deutetrabenazine and valbenazine as effective treatments for TD (evidence level A). In terms of other therapies, clonazepam and the herbal remedy Ginkgo biloba probably improve TD (evidence level B), while amantadine and tetrabenazine might be considered as TD treatments (evidence level C) (118). One issue that adds a further layer of complexity to our efforts to manage TD is the misperception that anticholinergics can be used to treat TD whereas they may in fact exacerbate it (140). This may have arisen from the misunderstanding and incorrect use of the term “extrapyramidal symptoms” for all drug-induced movement disorders, as mentioned previously, or the fact that anticholinergics were recommended for the management of “extrapyramidal symptoms” relating parkinsonism, although they were not intended for patients with TD. This highlights the need to improve the knowledge of clinicians who prescribe APDs regarding the phenomenology of drug-induced movement disorders and also the communication between psychiatrists and neurologists in the TD treatment pathway so that the most appropriate treatments will be given to patients.

In Asian countries and regions various therapeutic strategies for the management of established TD have been evaluated, including pharmaceutical agents and traditional remedies. Although deutetrabenazine and valbenazine are recommended in treatment guidelines for TD, they are not yet available in all Asian countries (Table 6). The efficacy and safety valbenazine has been evaluated in 256 Japanese TD patients in the phase II/III multicenter, randomized, double-blind, placebo-controlled J-KINECT study (141). The efficacy/safety profile of valbenazine was found to be consistent with that seen in original KINECT clinical trials undertaken in the USA, supporting its use for TD treatment in Japanese patients. Results showed a significant improvement from baseline in AIMS score at Week 6. The proportion of patients achieving ≥50% improvement of AIMS from baseline at Week 6 were 23.9, 47.2, and 10.3% in the 40-mg, 80-mg, and placebo groups, respectively.

**Table 6:** Availability of vesicular monoamine transporter 2 inhibitors in six representative East Asian countries.

**Drugs and countries**	**Valbenazine**	**Deutetrabenazine**	**Tetrabenazine**
Japan	✓	X	X
China	X	✓	X
South Korea	X	X	✓
Malaysia	X	X	✓
India	X	X	✓
Thailand	✓	X	X

A study in Taiwan evaluated switching treatment with an FGA to treatment with an SGA (aripiprazole at a dose of 5–30 mg/day) in patients who developed TD. Starting from week 2 of treatment with aripiprazole, AIMS total scores significantly decreased from baseline to the end of the study (142). A greater severity of TD and a lower severity of parkinsonism at baseline was both significantly associated with treatment response in this patient cohort.

A study in South Korea evaluated the use of kamishoyosan, an herbal medicine used for the treatment of parkinsonism and convulsion in traditional Chinese medicine, as a possible adjunctive treatment APD-induced tardive dyskinesia (143). The mean total AIMS scores of the 44 subjects with TD treated with kamishoyosan were found to have decreased significantly at weeks 4, 8, and 16. However, the design and validation of the study have been criticized and the results have not been replicated subsequently.

Ginkgo biloba leaves have been used in traditional oriental medicine for several 100 years and in recent times the leaf extract of Ginkgo biloba, EGb-761. Recently, EGb-761 has been evaluated in clinical trials as a symptomatic treatment for neurodegenerative disorders such as mild-to-moderate Alzheimer's disease and Parkinson's disease. A study in China found that EGb treatment of TD patients significantly decreased the total AIMS score compared with placebo at weeks 6 and 12 of treatment (144). As well as improving symptoms of TD, EGb-761 also increased the levels of brain-derived neurotrophic factor (BDNF)—which is suggested may have a role in the pathogenesis of TD—compared with placebo. The increase in BDNF in the EGb treated group was significantly associated with improvement in AIMS scores. It should be noted that these particular findings have not been subsequently replicated in other studies however EGb-761 is considered as having Class B evidence for its potential usefulness in improving TD symptoms according to the American Academy of Neurology classification (117).

Deep brain stimulation (DBS) has a level C evidence recommendation for severe, disabling and medically refractory TD (118), however brain surgery and device implantation should be considered carefully in someone with a major psychiatric disorder. The main sites with proven efficacy of stimulation are the subthalamic nucleus (STN) and internal globus pallidus (GPi). While DBS has proven effective and relatively safe in the treatment of TD and tardive dystonia, its usage is limited to Asian cohorts. A study from Japan by Koyama H et al. showed a significant improvement in mean Burke–Fahn–Marsden Dystonia Rating Scale (BFMDRS) scores at 1 month postoperatively of 75% with long-term improvement in BFMDRS scores of 78.0% (145). Another case report showed that pallidal DBS was an effective treatment for Chinese patients with tardive dystonia with an improvement in BFMDRS scores of 77% and in Global Dystonia Rating Scale scores of 66% (146). Although the effectiveness of DBS in TD and tardive dystonia is promising, further studies with DBS in other tardive syndromes are needed in Asian patients.

In summary, the primary treatment for TD should involve adjusting the medication that has been identified as a causative agent, or taking a VMAT2 inhibitor (if available) to suppress the abnormal movements if they are having a significant impact on the patient's quality of life. Research is ongoing to expand the range of treatment options and studies have been undertaken to evaluate the use of vitamins (e.g., vitamin E, vitamin B6) and/or supplements (e.g., melatonin, branched chain amino acids) to help manage TD. However, although the results from some small studies have been encouraging, more robust evidence is needed to support their widespread use in TD.

### 6.3 Patient awareness and knowledge of tardive dyskinesia

As the abnormal movements that characterize TD can be quite obvious to an observer, it is surprising that a high proportion of patients have a low subjective awareness of their symptoms. This lack of awareness of dyskinetic movement is not limited to TD patients but has also been observed in patients with Huntington's disease and in Parkinson's patients with levodopa-induced dyskinesias (147, 148).

A study from South Africa of 130 patients with schizophrenia who were experiencing TD found that 51% had no or low awareness of their condition (149). Similar findings have been reported in a study from Singapore of 607 schizophrenia patients which found that 67.4% were not aware of the presence of TD symptoms (46). In addition, awareness of the condition did not correlate with severity of TD symptoms. More recent studies, such as RE-KINECT, however, have revealed that when patients are asked specific questions in simple descriptive terms, over half report being aware of the abnormal involuntary movements of TD and feel they have a significant negative impact on their quality of life (111). These observations suggest that there is a need to improve knowledge and awareness of TD among patients at risk, so they can report symptoms they experience to their clinician and seek prompt treatment, and that focused discussions are needed between clinicians and their patients regarding their experience of TD symptoms.

As with many other medical conditions, patient support groups can be a valuable source of information and support, however there are few that are dedicated specifically to TD and in most cases it is incorporated under the wider umbrella of mental illnesses. In the US, the National Organization for Tardive Dyskinesia was established in 2019 which provides a range of resources including information on pharmaceutical and alternative treatments, Zoom TD support groups, and patients' personal stories via its website (www.tdhelp.org). A similar approach could be considered in Asian counties in local languages to help raise awareness of the condition and its need for treatment.

Clinicians can have an important role to play here in terms of directing patients at risk of developing TD, in particular those starting treatment with APDs, to key information about signs and symptoms to be aware of to allow early detection and management.

## 7 Discussion and conclusions

This systematic review provides an important perspective on the burden of TD in Asian countries and challenges in managing the condition that can help inform the overall global picture. The prevalence, presentation and management of TD various across the globe, so sharing information and experience from regional and nationally-focused studies can help highlight not only challenges, but also opportunities to optimize patient care.

We recognize that this review has multiple limitations, including the fact that the studies included in the analysis had heterogeneous designs and were often undertaken in only a small number of patients, so it is challenging to make comparisons and draw firm conclusions. Frequently, studies omitted details of inclusion and exclusion criteria, rendering it difficult to characterize patient cohorts. Additionally, evaluation methods vary widely, and studies may not take account that patients may switch between different APD medications. Importantly, the issue of fluctuations in TD symptoms is rarely addressed, and these can impact study results. Few studies have long-term follow-up periods that would enable full evaluation of the efficacy and safety of interventions.

Our analysis showed that, on balance, the prevalence of TD in Asia appears to be lower than in other world regions, although data are equivocal. If this trend is confirmed, it could indicate possible ethnic differences in TD prevalence or might reflect differences in patterns of APD use. However, the rise in APD use reported globally has also been observed in Asia, suggesting the number of patients at risk of TD is on the rise and will continue to present a healthcare challenge in the region. We know from studies in Asia that dose and duration of APD use are risk factors for TD, as seen in other world regions. However, considerable off-label and APD use and the use of higher than needed doses are contributing to the TD burden. While we now have some effective treatments for TD in the form of the VMAT2 inhibitors, deutetrabenazine and valbenazine, they are not available in all countries in Asia, and are also costly, which may limit widespread access. Consequently, the focus should be on minimization of TD risk. TD is a common occurrence in patients with psychotic disorders taking APD treatment, generally for many years, to control their symptoms, so it is essential that effective diagnostic and treatment pathways are in place. Currently, however, there is a lack of data on the long-term outcomes of the large proportion of patients who have TD but who need to remain on APDs to control their psychotic symptoms. One study of psychiatric inpatients who developed TD but remained on APDs found that, over a follow-up period of 14 years, their TD in fact improved (150).

**Figure 2:**
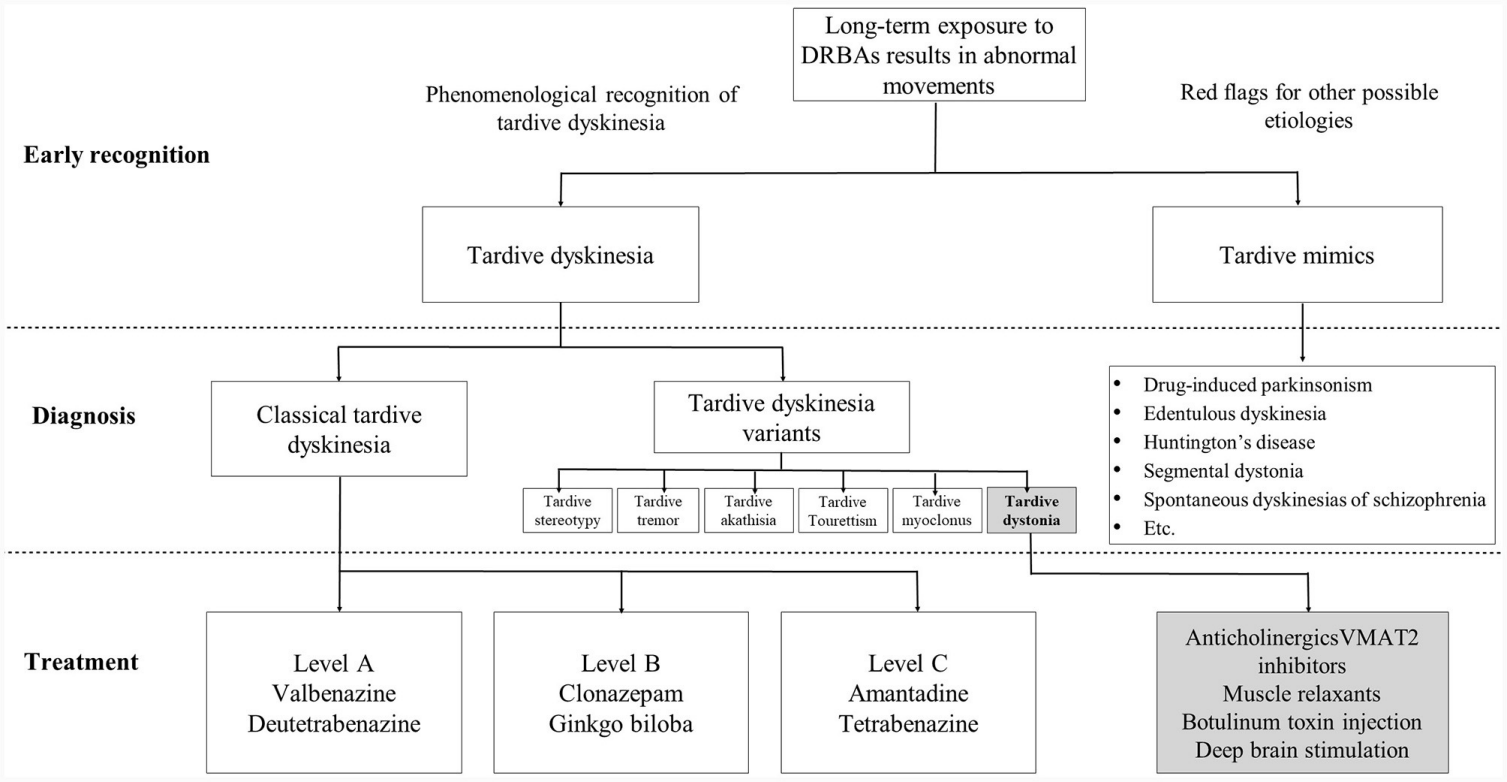
Early recognition and diagnosis of tardive dyskinesia (TD) and the ideal treatment pathway. Neurologists have an important role in the early recognition and differential diagnosis of TD, in particular, in recognizing movement disorder phenomenologies and identifying red flags for alternative diagnoses. For treatment of TD, neurologists should be included, wherever practical, as part of the management team alongside psychiatrists, working collaboratively and with joint decision-making to determine if suppression of abnormal movements is necessary and if specific treatment is needed. DRBAs, dopamine receptor blocking agents; VMAT2, vesicular monoamine transporter 2.

The literature review evaluated 85 studies from Asia and, of these, only two had been undertaken solely by neurology research teams, highlighting the very stark disconnect between the psychiatry and neurology disciplines, and the limited involvement of neurologists in TD research and treatment It is clear that neurologists can have a vital role in the diagnosis and treatment pathway for TD in Asia to provide much-needed movement disorders expertise, but this involvement is currently lacking. However, they need to be involved much earlier in the treatment pathway and not only once the condition has become serious and is impacting the patient's daily functioning (Figure 2). To achieve this, there is a need for greater collaboration between neurologists and psychiatrists, in particular where TD patients have serious psychiatric disorders where APD medication cannot easily be reduced or withdrawn. Awareness of and vigilance for TD symptoms should be a routine part of the clinical care of psychiatric conditions treated with APDs. Ideally, joint care pathways should be developed with an interdisciplinary approach to ensure early referral to movement disorders specialists for timely treatment when TD symptoms are suspected. It is recognized, however, that there is no “one-size-fit-all” approach to TD management and a multidisciplinary approach may not be possible across all clinical practice settings. Some psychiatric clinics may have limited access to movement disorders specialist or involving them may be cost prohibitive. In those cases, it may be more practical to reserve neurology consultation for patients with atypical, severe or treatment-refractory TD, or when underlying neurologic disorders are suspected. However, in an academic setting where neurologists with an interest in TD are available, joint clinics where neurologists and psychiatrists see TD patients together may be possible. A similar collaborate approach may also be possible in research settings in the field of TD where neurological manifestations of the condition need further exploration.

In terms of treatment options, it is important to define what therapies work best for particular TD phenotypes/phenomenologies and this requires the development of evidence-based treatment pathways reflecting different patient cohorts and ethnicities. There is still much we need to learn about the natural history of TD, its subtypes and potential risk factors, and how these vary across different world regions. Future research should involve collaboration between psychiatry and neurology teams, as well as considering regional differences in TD management practices that may be influenced by sociodemographic factors, resource challenges and inequalities in healthcare access.

## Data availability statement

The original contributions presented in the study are included in the article/Supplementary material, further inquiries can be directed to the corresponding author.

## Author contributions

RB: Conceptualization, Data curation, Formal analysis, Funding acquisition, Investigation, Methodology, Project administration, Resources, Software, Supervision, Validation, Visualization, Writing – original draft, Writing – review & editing. OP: Data curation, Formal analysis, Methodology, Validation, Writing – review & editing. H-FS: Writing – review & editing. TL: Writing – review & editing. JC: Writing – review & editing. PP: Writing – review & editing. HW: Writing – review & editing.
