# Supplementary material for: Tardive dyskinesia in Asia— current clinical practice and the role of neurologists in the care pathway
Source: Front Neurol. 2024 Feb 14;15:1356761. doi: 10.3389/fneur.2024.1356761 (PMC10901179; doi:10.3389/fneur.2024.1356761)
Supplement: Supplementary file 1 [file Data_Sheet_1.docx]

Supplementary Material

# Search methodology for tardive dyskinesia in Asia

## Data source and search

Literature about tardive dyskinesia in Asia were searched electronically in MEDLINE, Embase, and Scopus at the initiation of the project. The search keywords used were as follows: Tardive OR Tardive dyskinesia OR Tardive syndromes. Specifically, papers that were included in this review were required to have the term “Asia OR Asian” AND the above key words located within the title and/or abstract.

The study selection criteria and the selection process were as follows.

## Study selection criteria

The study was included in the analysis provided it fulfilled the following selection criteria. The study was conducted among patients who diagnosed with tardive dyskinesia OR tardive syndromes. The study contained data relevant to the study of tardive dyskinesia or tardive syndromes in Asia or Asian populations in several aspects including specific risk factors of development, epidemiology, causative agents, duration in onset after taking offending drugs (dopamine receptor blocking agents and others), awareness of symptoms, treatment algorithms, etc. The study was available in full length in English and published before the 4^th^ April 2023. Review articles, editorials, and clinical commentaries are excluded from the review process.

## Selection process (Figure 1 in manuscript)

### Titles and abstracts were identified from database searches and chosen with the selection criteria. Inclusion criteria includes the study about Tardive dyskinesia or tardive syndrome in Asia. Exclusion criteria included non-English literature, non-human subjects, and abstract unavailable literature.

### The reference lists of abstracts were chosen according to the selection criteria, and additionally relevant abstracts identified from a manual hand search were taken through stage 2 until no further abstracts were found.

### The chosen abstracts were retrieved as full-length articles, and only studies fulfilling the selection criteria were included as selected full-length articles.

### The finalized full-length articles fulfilling the inclusion criteria were identified for the systematic review process.

### Statistical analysis or meta-analysis was not attempted because of significant variability in study methodologies.

From 173 articles identified in the selection process, and subtraction of non-abstract publications, non-human subjects, non-English literature, and irrelevant studies within the scope of this review, at total of 82 articles were included in the analysis of tardive dyskinesia or tardive syndromes in Asia or Asian populations.
